# Editorial—Strain deposit and sequence deposit

**DOI:** 10.1007/s00253-024-13119-0

**Published:** 2024-05-27

**Authors:** Alexander Steinbüchel

**Affiliations:** Department of Molecular Microbiology and Biotechnology, University of Münster, Corrensstrasse 3, 48149 Münster, Germany

With this Editorial, I would like to contact the authors of the journal “***Applied Microbiology and Biotechnology***” and to provide arguments for the need to make the used strains and sequences available to colleagues.

AMB requests that the authors clearly define the strains if they have used bacteria, yeasts, fungi, or other microorganisms in a study that is described in a manuscript. The most direct way to ensure this is to provide the deposition number of the particular strain in a strain collection belonging to one of the about 150 strain collections of the **World Data Center for Microorganisms** (**WDCM**). Frequently used strain collections are ATCC, CBS, CCTCC, CGMCC, DSMZ, JMRC, NBRC, NCTC, NRC…… just to mention only a few examples.

On the homepage of the WDCM (https://gcm.wdcm.org), you will find excellent overviews and valuable information about this center. Strain deposits ensure that the strain is available from a high-quality collection which follows high standards and which ensures strain survival even in cases of accidents. AMB does not insist that all parallel strains are deposited if they were collected in a comparative study or during a screening; however, the most important and relevant strains should be deposited as examples.

As the authors should be aware from the journal submission guidelines (https://www.springer.com/journal/253/submission-guidelines#instructions%20for%20 Authors_Manuscript%20Preparation), availability must be enabeled by letting the readers know the deposition numbers in case of a new own isolate or of an already published strain. Such numbers describe the microbial strain unambigously, and misunderstandings are almost excluded.

The physical strain deposit **cannot** be replaced by a deposit of a sequence of the 16S-RNA gene in case of prokaryonts or a sequence of the 18S RNA gene in case of eukaryonts. The deposition of these sequences in a recognized sequence data bank (preferred repositories: GenBank, EMBL, DDBJ) and the provision of the accession number in the “Materials and Methods” section will be in addition necessary if the used strain is a new isolate that was never used before.

The deposition of genome sequence data or the sequences of particular genes should be handled in the same way as described above. This ensures that the genes are clearly defined!

Furthermore, the strain repositories check in most cases the correct affiliation of the deposited strains, and there is also the tendency to unravel the partial or even the complete genome sequence.

All this makes sure that the used strain is unambigously defined. This will also allow others to build up on the published study. If your studies are economically relevant, you will probably apply for a patent before the manuscript is submitted and published; concomitant with the application, the relevant strain has to be deposited according to the Budapest Treaty, and the strain is released by the depository only under very controlled conditions. If the strain is released to others by the authors, it is evident that the authors acknowledge the receipt of the strain and that they refer to the publication.

The authors are kindly asked to follow the guidelines regarding strain deposit carefully and accurately. This will allow a fast transition of the manuscript upon submission to peer review and make the data much more reliable for the scientific community.

Prof. Dr. Alexander Steinbüchel

(Editor-in-Chief) 

